# Scheduling medicines as controlled substances: addressing normative and democratic gaps through human rights-based analysis

**DOI:** 10.1186/s12914-020-00231-1

**Published:** 2020-04-21

**Authors:** Diederik Lohman, Damon Barrett

**Affiliations:** 1 Dornsife School of Public Health, Drexel University, Nesbitt Hall, 3215 Market Street, Philadelphia, PA 19104 USA; 2 School of Public Health and Community Medicine, Institute of Medicine, University of Gothenburg, Box 453, 405 30 Gothenburg, Sweden

## Abstract

Recent years have seen contentious debate about efforts to schedule medicines such as ketamine and tramadol under the international drug control conventions. Proponents argue that misuse poses a significant risk to public health and that scheduling would help address these problems. However, scheduling of medicines can negatively affect their availability, accessibility and affordability for medical purposes, with serious health consequences for patients, especially in low and middle-income countries. The current process for scheduling medicines under the international drug control conventions does not provide sufficient normative standards through which balanced decisions may be reached. It is undemocratic in its structure and opaque in its reasoning. In this article, we argue that such decisions represent de facto limitations on the right to health and may engage the principle of non-retrogression. Using the examples of ketamine and tramadol, we propose that standard legal tests in international human rights law can help to address the normative and democratic deficits in the system and produce more rigorous, fairer and more transparent decisions.

## Background

In 2015, the regulation of the anaesthetic ketamine sparked heated debate at the UN Commission on Narcotic Drugs (CND) in Vienna, Austria. The CND is the political body in the United Nations tasked with, among other things, decisions as to whether to bring new substances under the controls set out in the three UN drug control conventions (to which we return below). China had requested a vote during CND’s annual meeting on scheduling ketamine under the 1971 Convention on Psychotropic Substances, the treaty under which substances such as MDMA (3,4-Methylenedioxymethamphetamine**)** and LSD (Lysergic acid diethylamide) are controlled [1]. It argued that ketamine had become a widely abused drug in China and elsewhere in the region and was causing significant public health harms. Placing the medication under international control, China argued, was essential to countering these harms [2]. On the other side, various medical associations and civil society groups argued that putting ketamine under international control would severely restrict medical access to a safe anaesthetic that is widely used in surgeries in low and middle-income settings and for which there is no inexpensive alternative [3–6]. They contended that many people might lose access to potentially life-saving surgeries if ketamine was scheduled, or would have to undergo them without proper anaesthesia. On this occasion, after much back and forth, China asked for the vote to be cancelled after it became clear that its proposal didn’t have enough support to schedule the medicine [2].

The issue at the heart of this debate, however, remains very much alive. Various medicines are not currently scheduled as controlled substances even though they have psychoactive properties that can trigger the attention of the international drug control system. The nonmedical use of some of these medicines is not uncommon. For example, the World Health Organization Expert Committee on Drug Dependence (ECDD) has in recent years reviewed not just the misuse potential of ketamine [7–9], but also of tramadol [10–13], an opioid analgesic, etizolam, a sedative [14, 15], and pregabalin, a neuropathic pain reliever [16, 17]. The latest WHO review of tramadol, which recommended against international scheduling, elicited a harsh rebuke from Egypt, the country that is leading the push for its scheduling, as well as from China and Nigeria [18]. For some time concerns have been raised about non-medical use of ketamine and tramadol by the International Narcotics Control Board (INCB, which oversees the implementation of the UN drug control conventions) [19–26] and UN Office on Drugs and Crime (UNODC, part of the UN secretariat) [27–31] Both have suggested in strong terms that international scheduling is the solution but stopped short of explicitly and publicly recommending it.

This question is likely to keep rearing its head and generating controversy. These seemingly remote decisions in Vienna have very real consequences for medical practice and public health. While nonmedical use is a serious problem, it is also clear that national and international scheduling leads to reduced availability and accessibility of medicines [32–43], may negatively affect perceptions of the medicine (creating a ‘chilling effect’ on prescribing) [36–40], and may drive up cost [41].

This article provides an overview of the current system for placing substances under international control, which is set out in the relevant treaties, and involves technical review by the WHO and a political vote at the UN Commission on Narcotic Drugs. We demonstrate that the process has both normative and democratic deficits. These deficits interfere with an appropriate consideration of the real-life benefits and unintended consequences of scheduling, with striking an appropriate balance between the interests of ensuring access to medicines and limiting the harms associated with their nonmedical use. They also result in a lack of explicit and sufficient justification for any decisions ultimately taken, which is key to transparency.

We introduce the relevant human rights norms applicable to this issue and present a human rights-based test to be applied to international scheduling, grounded in the right to health. This, we argue, would address an important normative deficit in the current process and ensure careful examination of the potential effects and consequences of scheduling a specific medicine based on shared principles. Moreover, in demanding sufficient ‘reason-giving’ it would make the process more compliant with basic principles of accountability and transparency that are central to good governance and global administrative law [44]. Consistent with the principle of ‘non-retrogression’ in international human rights law, we propose a presumption against scheduling medicines as internationally controlled substances until such a test has been met.

## The scheduling system under international drug control law

International drug control is governed by three multilateral treaties: the Single Convention on Narcotic Drugs 1961, as amended by its 1972 Protocol (‘Single Convention’) [45], the Convention on Psychotropic Substances 1971 (‘1971 Convention’) [46], and the Convention against Illicit Traffic in Narcotic Drugs and Psychotropic Substances 1988 (‘Trafficking Convention’) [47]. The Single Convention primarily controls plant-based substances and their derivatives, in particular coca, cannabis, opium poppy. The 1971 Convention controls synthetic substances such as LSD, MDMA and amphetamines. The Trafficking Convention focuses on penal measures and transnational law enforcement relating to these substances and precursor chemicals. The system builds upon the general obligation, set out in Article 4(c) of the Single Convention: ‘ … to limit exclusively to medical and scientific purposes the production, manufacture, export, import, distribution of, trade in, use and possession of drugs’. [45] This reflects the dual aims of both suppressing the illicit market, while ensuring access to controlled medicines.

Central to the system are lists of substances set out in various ‘schedules’ according to their risk profile (Article 2, Single Convention, Article 21,971 Convention. See Hallam et al. 2014 [48]). Under the Single Convention there are four schedules, with schedules I and IV being the most restrictive. Substances on schedule I may also be placed on schedule IV if they are particularly risky and do not have significant medical value. Under the 1971 Convention, schedule I is the most restrictive. Once placed on these schedules, specific treaty obligations and domestic regulatory provisions apply, with important effects in terms of prescription, import and export contols. For example, in some countries domestic regulations impose highly burdensome procedures for procurement or stocking of scheduled medicines or arbitrarily restrict prescribing and dispensing of these medicines. Article 21 of the Single Convention imposes specific limitations on quantities that may be manufactured and imported. If placed on schedule I of the Single or 1971 Conventions, all of the controls in those conventions apply.

Decisions whether or not to place substances under international control are made by majority vote at the CND. Under Article 3 (1) of the Single Convention a State party or the WHO may notify the Secretary-General of the need for a change to the schedules (i.e. the inclusion of a substance, its removal, or moving it from one schedule to another). The Secretary-General then brings this to the attention of the CND or to the WHO if the issue was raised by a State party. If the WHO finds that a substance is ‘liable to similar abuse and productive of similar ill effects’ to substances already in schedules I and II, it must communicate its finding to the Commission which ‘may’ decide to schedule the substance (Article 3 (3)(iii)).

As the official commentary to the Single Convention states, substances may only be scheduled if they produce similar effects to those already scheduled. In practice this is wide ranging, relating to anything ‘similar’ to cannabis, cocaine or opiates [49]. The WHO decision is based on a study of its Expert Committee on Drug Dependence (ECDD), to which we return below. If the WHO finds that a substance ‘is particularly liable to abuse and to produce ill effects’ and this ‘is not offset by substantial therapeutic advantages’, the Commission ‘may’, place that substance in schedule IV, the most stringent (Article 3 (5)). Pursuant to Article 3 (7) of the Single Convention, the UN Economic and Social Council (ECOSOC) may ‘confirm, alter or reverse the decision of the Commission [on Narcotic Drugs]’, and the Council’s view is final. In practice it has never reversed CND. The CND’s decisions are communicated to the Secretary-General, the WHO and States parties to the Convention and, critically, ‘become effective with respect to each Party on the date of its receipt of such communication’ (Article 3 (7)).

The process under the 1971 Convention is broadly similar in its process, but there are important differences. In particular, unlike the Single Convention, the 1971 Convention requires a two-thirds majority, imposing a higher burden (Article 17). Moreover, according to Article 2 (5) the role of the WHO is explicitly ‘determinative as to medical and scientific matters’, and States are expressly allowed to take into account other relevant ‘economic, social, legal, administrative and other factors’. These important considerations of voting, technical review, and taking into account wider factors, however, all speak to deficiencies in the system, to which we now turn.

## The normative and democratic deficits in the scheduling system

The normative deficit in the system relates both to the WHO analysis and decision-making by CND Member States. The instructions for the ECDD, which conducts the review of substances and makes recommendation on international scheduling, are illustrative [50]. A 2010 WHO guidance document states that the ECDD should provide a summary assessment of its findings on ‘the extent or likelihood of abuse, the degree of seriousness of the public health and social problem, and the degree of usefulness of the substance in medical therapy, together with the advice on the control measures, if any, that would be appropriate in the light of its assessment.’ Thus while the need for a balance is implied, any discussion of *how* to balance potential control measures with medical availability is missing. The documentation WHO prepares for the ECDD’s review does not include an assessment of the potential impact of international scheduling for the availability, accessibility or affordability of medicines containing the substance, or any information on the potential effectiveness of control measures short of international scheduling [12, 16].

In practice the ECDD does consider the question of when a substance has important medical applications. After all, how else would it be able to reach a recommendation on control measures? For example, in the most recent review of tramadol, the ECDD recognised that the substance poses significant health risks but recommended against international scheduling because of its medical importance [16]. In the 2012 review of ketamine, it concluded that ‘that if ketamine were placed under international control, this would adversely affect its availability and accessibility. This in turn would limit access to essential and emergency surgery, which would constitute a public health crisis in countries where no affordable alternative anaesthetic is available.’^8^ However, WHO’s guidance does not offer a mechanism for weighing the dual risks posed by the substances themselves and the potential decision to schedule it. For example, it is unclear what criteria were used to reach its decisions on tramadol and ketamine, which is problematic from a transparency and accountability perspective. It also made it easier for countries such as Egypt and Nigeria to attack the ECDD’s recommendation.

After the WHO review, a decision must be taken in the political body – the CND. While, as outlined above, the drug control conventions include some criteria for scheduling substances based on the public health risks they pose, they are silent on how the public health risks posed by the substances should be weighed against the risks posed by international scheduling. And while the 1971 Convention allows for ‘economic, social, legal, administrative and other factors’ to be considered, it is silent as to what role they play and how to balance them. It is a discretionary provision, not a normative test for reasoned decision making. No guidance on questions of international scheduling exists for the CND beyond the conventions and their official commentaries, which are insufficient for addressing the above concern.

Turning to the democratic deficit, once a recommendation from the WHO reaches the CND, a small minority of UN members can make decisions that are binding on all States parties. Under the Single Convention, a simple majority of the 53 Member States of the CND can add a substance, such as tramadol, to an international schedule. Thus, 27 states can engage international legal obligations for all 180 states that are party to the Single Convention and the related obligations under the Trafficking Convention. Under the 1971 Convention, which requires a two-thirds majority, 36 states can schedule a substance, such as ketamine, internationally. Countries do not have any possibility under the conventions to opt out of a scheduling decision. In addition, approximately 70 countries do not have permanent diplomatic representation in Vienna and thus face significant barriers to direct participation in both informal discussions and debates and the votes themselves [51].

The normative and democratic deficits are related. In particular, the normative gap exacerbates the democratic deficit based on the voting rules by allowing for decisions to be taken by the small minority, without sufficient reasons being given. It is well recognised that governance mechanisms with legal functions should, in the interests of transparency and legitimacy, provide sufficient reasons for any decisions taken [44]. The issue is that the scheduling process at the CND is insufficient in terms of reason-giving for the legal nature of the decisions being taken. The normative deficit essentially obscures the reasons for decisions from States that will have to bear international legal obligations based on them, and from those that would be affected on the ground. The democratic deficit due to the voting procedure ultimately requires treaty amendment in order to be repaired. In the meantime, however, adequately supported justifications for scheduling decisions should be shared and subjected to diplomatic scrutiny prior to the vote, and afterwards at ECOSOC, where such decisions may be officially reviewed.

## The relevance of international human rights law and the right to health

Almost every State in the world has agreed to be bound by at least one human rights treaty that recognises the right to health. Article 12 of the International Covenant on Economic Social and Cultural Rights (ICESCR), with 169 States parties, guarantees a right to the ‘enjoyment of the highest attainable standard of physical and mental health’ [52]. Similarly, Article 24 the UN Convention on the Rights of the Child (CRC), with 196 parties, guarantees the child’s right to health (Only the United States has not ratified the CRC) [53]. Indeed, scheduling decisions of the CND directly engage CRC obligations. Article 33 requires that States protect children from ‘narcotic drugs and psychotropic substances as defined in the relevant international treaties’. Thus, as substances are scheduled by a vote of the CND, they are folded into the scope of Article 33. As the CRC must be read holistically, this carries with it the wider obligations of the convention, including the right to health under Article 24 [54].

The Committee on Economic, Social and Cultural Rights (CESCR), the UN body charged with monitoring compliance with ICESCR, has provided what remains the most authoritative statement of the right to health in international law. In its General Comment No. 14, issued in 2000, it stated that States parties must ‘refrain from interfering directly or indirectly with the enjoyment of the right to health’, which includes interfering with existing health services (para 43) [55]. Ketamine (for anaesthesia), tramadol (for pain) and pregabalin (for neuropathic pain and other conditions), are all widely used medicines, including in low and middle income countries. Scheduling a medicine as a controlled substance constitutes an interference because it results, by design, in the application of additional rules and restrictions to the medicine, which may impede existing access to it. Scheduling existing medicines therefore engages the principle of non-retrogression, which involves a strong presumption against backward steps [55]. In this case, scheduling may constitute retrogression if the medicine becomes less available or accessible. Outside of economic shocks, natural disasters or conflict, and other factors beyond the State’s control, the CESCR construes the principle of non-retrogression strictly. For any deliberate measures amounting to retrogression ‘the State party has the burden of proving that they have been introduced after the most careful consideration of all alternatives and that they are duly justified by reference to the totality of the rights provided for in the Covenant’ [55].

It must also be noted that access to essential medicines is considered a ‘core minimum’ requirement of the right to health, adding further to the above requirements to consider right to health implications [55, 56]. Ketamine, for example, is already on the WHO Model Essential Medicines List [57]. While tramadol is not currently on the list it is included on WHO guidelines for cancer pain relief and is on multiple national essential medicines lists [58].

The right to health is not absolute, however, and it is recognised that, as with other questions of public health ethics, some balancing between the right and other considerations may be needed. According to Article 4 of the ICESCR, ‘the State may subject such rights only to such limitations as are determined by law only in so far as this may be compatible with the nature of these rights and solely for the purpose of promoting the general welfare in a democratic society.’ [52] Such limitations clauses are common in human rights law. From them well accepted tests for compliance have developed. This is where there may be real added value for decisions around scheduling. Measures amounting to limitations on existing health services or goods must pursue a *legitimate goal*, and *be potentially effective*, *based in law*, and *proportionate* to the legitimate goal, considering the impact of such measures on the totality of the rights affected. The CESCR has explicitly related this test to Article 4 of the Covenant [55]. The test may be seen in steps. If there is no legitimate goal, the measure fails at the first hurdle. If it is not based in law, it does not matter if it might be effective. And if it is not potentially effective, then any infringement on rights it entails is by definition disproportionate to its purported aim, because that aim cannot be met.

Combining this test with the presumption against retrogression, we argue that there should be a presumption against scheduling medicines unless this test has been passed. This complements the recommendations made by others, challenging a ‘regulatory panic’ in the regime, alongside institutional and political biases that lead to the overinclusion of substances [48]. Moreover, these standards should be taken on board both by the WHO ECDD and CND Member States. This, we suggest, helps to alleviate the normative deficits identified above. With regard to the legitimacy of including such a test in the scheduling process, for the WHO, the right to health is contained in its own Constitution [59]. The UN Commission on Narcotic Drugs is a body established under the Charter of the United Nations, which itself has, as one of its three primary aims, the advancement of human rights (articles 1, 55, 56) [60]. Moreover, while the CND operates collectively as a commission, each individual Member State must give its own reasons for its vote. Every CND Member State bar the United States has its own right to health treaty obligations that do not cease to operate in the CND’s boardroom. We would add that nothing in the drugs conventions or elsewhere precludes such a test being employed in a consistent and official manner, while basic principles of good governance require attention to these deficits.

## Applying right to health standards to the scheduling process

While in some places ketamine, tramadol or pregabalin are already scheduled as controlled substances under national law [9, 12, 14], in most countries they are designated as prescription medicines—that is, pharmacies may dispense them only when presented with an appropriate medical prescription. As international controls are explicitly intended to place restrictions on both the availability of the medicines (by imposing international controls on their production and movement) and their accessibility (through related prescription requirements and tougher regulations) scheduling constitutes an interference with the right to health and a form of retrogression. Thus, the presumption against retrogression in human rights law applies.

This presumption is rebuttable if scheduling meets the legality, effectiveness and proportionality test outlined above. Let us consider these criteria in turn.

### Legitimate goal requirement

Is the objective of the interference or retrogression justifiable? In this case, the reason governments propose the international scheduling of a medicine is to counteract their non-medical use and the associated health risks. This is clearly a legitimate objective. Indeed, states have an obligation under international human rights law to take effective steps to protect the people in their jurisdiction from threats to public health and human rights. In case a substance—whether that is a toxic chemical or a psychoactive substance—causes or may cause significant health harms, a state has a human rights duty to minimise that harm, which may include placing restrictions on its production, sale and use [55].

### Legality requirement

If the proper legal procedure for scheduling as outlined in the international drug conventions is followed this requirement will be met. However, there is the risk that this basic requirement might not be met. In 2016, China sought a vote to schedule ketamine even though WHO had recommended against it, which could have resulted in a failure to meet the right to health test [1].

### Effectiveness requirement

States must be able to make a compelling argument that scheduling is a potentially effective measure to achieve the legitimate goal. This requires some reflection on the strategies the drug control conventions entail. While intuitively it may feel correct to assume that scheduling a substance would be effective in reducing its nonmedical use and thus health harms, decades of experience suggests a rather more complex and uncertain picture. There is little doubt that international scheduling has a significant impact on licit production of medicines placed under international control. After all, pharmaceutical companies and distributors have no choice but to operate within existing international and national legal frameworks. To produce medicines they require relevant pharmaceutical licenses; to distribute and sell their medicines they must obtain registration from relevant authorities, etc. International scheduling adds an additional layer of bureaucratic requirements that these companies must comply with if they want to continue to produce and sell these medicines. Research on the availability, accessibility and affordability of morphine and other internationally controlled opioid analgesics shows that these extra requirements result in companies stopping to produce or distribute these medicines or increasing their price exactly because they need to comply with additional requirements [35, 40–42].

The same is not true for illicit production and sale of controlled substances, which by definition operates outside legal frameworks. Indeed, there are large questions about the effectiveness of scheduling as a tool to counter production, trafficking and sale of illicitly produced controlled substances. This is most clearly illustrated by the fact that substances such as heroin, cocaine, and marijuana, which have been under international control for more than half a century, continue to be widely available in many places despite hundreds of billions of dollars spent on interdiction [28, 43].

This distinction between the effects of scheduling on legally and illegally produced substances is significant. Critically, for both ketamine and tramadol it appears that the substance that is often misused in low and middle-income countries is not actually the officially registered medicine but an illegally produced or imported substance that is fraudulently packaged and sold as ketamine or tramadol [27, 28, 61]. Such products meet WHO’s definition of falsified medical products [62] and the producers or smugglers of these products already operate outside the law. The production and sale of falsified medical products is illegal in most jurisdictions [63]. Although legal frameworks for addressing falsified medicines at the national and international are widely criticized as deficient [64], generally national customs officers do already have the authority to stop these products from entering the country; law enforcement officers already have the authority to counter their distribution and sale to customers; and law enforcement officers in producer countries can shut down manufacturing of these products. The primary problems therefore appear to be the inadequate enforcement of existing laws and regulations on falsified medicines, rather than the absence of legal tools to act. This raises key questions about the effectiveness of international scheduling, which creates additional legal tools—and imposes an additional bureaucratic burden for legitimate medicines—but does not change enforcement of existing laws.

The situation in countries that have already placed tramadol on national schedules suggests that this measure has limited effect on non-medical use. In Egypt, for example, non-medical use of tramadol appears to continue to be a significant problem today even though the government scheduled it under national law in 2013 [65–67]. At the same time, doctors report that the legally produced and registered medication tramadol has become increasingly difficult to obtain, even upon presentation of a medical prescription, and that the medication has become stigmatised [65].

### Proportionality requirement

The principle of proportionality holds that when a state puts in place measures that interfere with the enjoyment of the right to health, it must use the least burdensome or invasive measure to achieve the goal. Further, it must ensure an appropriate balance between the different interests at stake: Reducing public health harms due to non-medical use of the substance and ensuring that patients with a legitimate need for medicines containing that substance have adequate access to them. The proportionality requirement is particularly important in the case of international scheduling because of the large body of research on the negative effects in terms of availability and accessibility afterwards [32–43]. Any decision to schedule a medicine internationally carries a significant risk to patients who need the relevant medicine, especially in low and middle income countries.

It is thus imperative that decision makers consider all available options for addressing the public health risks posed by a substance, and settle on the least invasive one that can be effective. For example, in some countries, tramadol is legally a prescription medicine but frequently sold over-the-counter in pharmacies to any customer able to pay as pharmacy staff simply ignore the prescription requirement [36, 68]. In this scenario, while international or national scheduling may be effective at preventing non-medical use of tramadol, such measures will likely fail the proportionality test because it is far more invasive than another measure that may also be effective: enforcing existing prescription requirements. Similarly, where tramadol or another medicine is not legally categorised as a prescription medicine and can thus be legally sold over-the-counter, countries have the option of changing national law or regulation to require a prescription for their sale without the need for bringing the medicine under international control.

Ultimately, the decision whether or not to schedule internationally will remain a judgment call that reasonable people may feel should go different ways. However, at present, the normative deficit in the system could allow for a medicine to go through review at WHO and a vote at CND without these kinds of issues arising other than in an ad hoc fashion. Nor would it require transparent reasons to be given as to how such issues were addressed in reaching a decision. The test we propose requires a transparent deliberative process of weighing the pros and cons of different options before making that decision, and in doing so requires that reasons are given for scheduling decisions, rooted in shared human rights-based standards.

## Conclusion

International scheduling of existing medicines puts in conflict two important public health objectives: protecting people from the health harms certain substances may pose and ensuring the adequate availability, accessibility and affordability of medicines that contain those substances. It also engages potentially competing drug control and human rights obligations, at a time when human rights are becoming more and more mainstreamed in international drug policy discussions, and system-wide coherence within the SDG framework is a central concern [69, 70]. The current process for international scheduling of medicines has an important normative deficit in the absence of shared principles of decision making to weigh these interests. It has a related democratic deficit given such decisions can be made by a minority of States creating legal obligations for all States parties. A human rights-based legality, effectiveness and proportionality test can help address these deficits and bring the decision-making process more into line with good governance standards.

## Data Availability

Not applicable.

## Abbreviations

CESCR: UN Committee on Economic, Social and Cultural Rights
CND: UN Commission on Narcotic Drugs
CRC: UN Convention on the Rights of the Child
ECDD: WHO Expert Committee on Drug Dependence
ICESCR: International Covenant on Economic Social and Cultural Rights
INCB: International Narcotics Control Board
LSD: Lysergic acid diethylamide
MDMA: 3,4-Methylenedioxymethamphetamine
UNODC: UN Office on Drugs and Crime
WHO: World Health Organization
